# Multi-institutional application of Failure Mode and Effects Analysis (FMEA) to CyberKnife Stereotactic Body Radiation Therapy (SBRT)

**DOI:** 10.1186/s13014-015-0438-0

**Published:** 2015-06-13

**Authors:** Ivan Veronese, Elena De Martin, Anna Stefania Martinotti, Maria Luisa Fumagalli, Cristina Vite, Irene Redaelli, Tiziana Malatesta, Pietro Mancosu, Giancarlo Beltramo, Laura Fariselli, Marie Claire Cantone

**Affiliations:** Dipartimento di Fisica, Università degli Studi di Milano, Via Celoria 16, Milan, 20133 Italy; Fondazione IRCCS Istituto Neurologico Carlo Besta Milano, UO Direzione Sanitaria, Milan, Italy; Centro Diagnostico Italiano, Reparto Cyberknife, Milan, Italy; Ospedale San Giovanni Calibita Fatebenefratelli, UOC Fisica Sanitaria AFAR, Rome, Italy; Reparto di Radioterapia Oncologica, Istituto Clinico Humanitas, Milan, Italy; Fondazione IRCCS Istituto Neurologico Carlo Besta Milano, Unità di Radioterapia, Milan, Italy; Present address; Now at: Clinica Luganese, Lugano, Switzerland

**Keywords:** FMEA, SBRT, CyberKnife, Tracking, Liver, Spine

## Abstract

**Background:**

A multidisciplinary and multi-institutional working group applied the Failure Mode and Effects Analysis (FMEA) approach to assess the risks for patients undergoing Stereotactic Body Radiation Therapy (SBRT) treatments for lesions located in spine and liver in two CyberKnife® Centres.

**Methods:**

The various sub-processes characterizing the SBRT treatment were identified to generate the process trees of both the treatment planning and delivery phases. This analysis drove to the identification and subsequent scoring of the potential failure modes, together with their causes and effects, using the risk probability number (RPN) scoring system. Novel solutions aimed to increase patient safety were accordingly considered.

**Results:**

The process-tree characterising the SBRT treatment planning stage was composed with a total of 48 sub-processes. Similarly, 42 sub-processes were identified in the stage of delivery to liver tumours and 30 in the stage of delivery to spine lesions. All the sub-processes were judged to be potentially prone to one or more failure modes. Nineteen failures (i.e. 5 in treatment planning stage, 5 in the delivery to liver lesions and 9 in the delivery to spine lesions) were considered of high concern in view of the high RPN and/or severity index value.

**Conclusions:**

The analysis of the potential failures, their causes and effects allowed to improve the safety strategies already adopted in the clinical practice with additional measures for optimizing quality management workflow and increasing patient safety.

## Background

Stereotactic body radiation therapy (SBRT) was introduced several years ago but has become only recently a recognized treatment option for many anatomical sites [1–7]. SBRT delivers high radiation doses to small lesions with short fractionation schemes under the most stringent conditions, allowing high dose conformity and sparing of healthy tissue. This should help to overcome the long-term toxicity concerns of conventional radiation therapy (RT). To date, different techniques exist to deliver SBRT, all sharing a number of common properties: an ensemble of convergent beams or arcs is used to target a circumscribed, well defined lesion [3].

A characteristic aspect is the high level of complexity of all these methodologies, which places demands of innovative approaches to patient safety. Indeed, potential SBRT-related errors could lead to severe injury to the patients in view of the high radiation dose delivered per single fraction. In this context, proactive methods of risk analysis, aiming to anticipate the potential hazards that may occur during the RT process, are particularly fit to investigate the risks of this clinical practice. In this scenario, the Failure Mode and Effects Analysis (FMEA), routinely employed in high-risk industry, is an emerging and effective actor, recognised as a powerful and increasingly popular tool for proactive risk analysis in modern radiation oncology [8–21]. Furthermore, in order to address the prevention of accidental and unintended medical exposure, proactive risk management approaches in RT are recently required by the new European Union Basic Safety Standards [22].

As far as the authors know, only few papers dealing with the application of the FMEA approach in stereotactic radiation therapy are currently available in the literature. Perks and colleagues [17] analysed the SBRT process performed on patients with abdominal compression to limit diaphragm motion. Masini and colleagues [14] performed risk analysis for intracranial stereotactic radiation surgery practices. Similarly, Younge and colleagues tested the practical implementation of the FMEA in the design of a quality assurance program for stereotactic radiosurgery treatments [21]. In all these cases, the RT processes involved the use of conventional linear accelerators. When radiosurgery dedicated machines equipped with an online tracking system both for static and moving lesions are considered, as in this study, additional specific safety measures have to be evaluated. Such machine is CyberKnife® (Accuray Inc. Sunnyvale, USA), a robotic image-guided frameless stereotactic system used for irradiation of both cranial and extracranial regions [23–31].

In this study the FMEA approach was applied to assess the risks for patients undergoing SBRT treatments for lesions located in spine and liver in two CyberKnife® Centres with ten years of experience. The various sub-processes characterizing the SBRT treatment were identified to generate the process trees of both the treatment planning (taking into consideration the steps following target and critical organs delineation) and delivery phases (in particular including the tracking steps). This accurate analysis of the RT process drove to the identification and subsequent scoring of the potential failure modes, and finally to the suggestion of novel solutions aimed to increase patient safety.

## Methods

### SBRT with Cyberknife®

The specific SBRT processes implemented at the CyberKnife® Centre, Centro Diagnostico Italiano (CDI), Milan, and at Carlo Besta Neurological Institute Foundation IRCCS (Besta), Milan, were considered for the analysis. This study was focused on SBRT treatments of liver (CDI) and spine lesions (Besta), using fiducial markers coupled with Synchrony® Respiratory Tracking System (SRTS) and Xsight® Spine Tracking System (XSTS) for target localization, respectively. Common feature of the SBRT protocol for both the Cyberknife® Centres was the preliminary acquisition of computed tomography (before and after contrast medium injection if required, slice thickness 1 mm, 15 cm extension below and above lesion site) and magnetic resonance imaging (before and after contrast medium injection if required, slice thickness 1.5-2 mm). The CT for liver treatment was acquired during a single breath-hold for each of the three vascular phases.

The images acquired with different modalities were then fused and used for accurate delineation of target and organs at risk (OARs) contour. The radiation treatment was then planned on the CT images without contrast medium with single or multiple fraction schedules (usually 3, 15Gy/fraction for the liver case and usually 1 to 5 for the spine case, dose range 12-25Gy).

The CDI CyberKnife centre is equipped with two Cyberknife® VSI™ 9.6 systems (6 MV X-ray beam, dose rate 800MU/min and 1000MU/min, Multiplan® 4.6 treatment planning system (TPS) and MD Suite administrative application). The Cyberknife Unit staff includes 2 radiation oncologists, 4 radiation therapists, 3 medical physicists and 2 office workers. The centre treats about 800 patients a year, equally distributed among cranial and body lesions.

Liver lesions in particular are treated with the SRTS system [32] intended to enable dynamic image-guided stereotactic radiotherapy of targets moving under the influence of respiration. This system synchronizes beam delivery with the target motion by building a model (Synchrony model), constantly relating target position and thorax respiratory motion of the patient. In particular, LED markers are positioned on the patient’s thorax (specifically on a Synchrony vest worn by the patient). LED motion is then detected by a camera array and used to determine breathing waveform. Meanwhile, the target position is assessed by the fiducial markers position on the live X-ray images. Basing on the Synchrony model built, the treatment manipulator adjusts and compensates for the necessary movements to ensure an accurate treatment.

The radiation equipment of the Besta radiotherapy department consists of two 6 MV X-ray beam treatment machines: an Elekta Synergy® linear accelerator (dose rate 300 MU/min, XiO Treatment Planning Systems, Mosaiq Record & Verify) and a Cyberknife® system 9.6 (dose rate 1000 MU/min, Multiplan® 4.6 TPS, Multiplan MD Suite administrative application). The staff collaborating with the radiotherapy department consists of 4 radiation oncologists, 5 radiation therapists, 3 medical physicists (2 dedicated) and 2 office workers. On the whole, patients treated with the two machines amount to an average of 700 per year, mainly including cranial and spine lesions, but only the Cyberknife® system is used for SRT (380 patients per year).

For this system, treatment adaptation to target motion for spine lesions is ensured by XSTS [32]. This non-invasive system registers non-rigid and bony anatomy landmarks to automatically locate and track tumours, eliminating the need for surgical implantation of fiducials. In particular, a region of interest (ROI) containing an 81-node grid is defined when the treatment plan is created. During treatment delivery the XSTS computes target displacement by monitoring the displacement of nodes of the ROI in the live X-ray images relative to the nodes in the Digitally Reconstructed Radiograph (DRR) images. XSTS is eligible for nearly 100 % of the spine cases and, as in the case of SRTS, allows for the treatment manipulator to adjust and compensate for target movements.

Considering all the peculiarities and complexities of the tracking systems described for target localization, ample space is given to the related potential failure modes analysis.

### Failure Modes and Effects Analysis (FMEA)

The FMEA was carried out by a multidisciplinary and multi-institutional team composed by specialists in the SBRT process (medical physicists, radiation oncologists, radiation therapists), under the supervision of experts in risk analysis.

The first step of the FMEA consisted in the process tree generation, through the identification of all the sub-processes involved in the stages of interest:

- treatment planning following target and OARs delineation, common in most of the aspects at both the Centres involved in the study [33];
- treatment delivery to liver tumours by using fiducial markers coupled with SRTS. This stage was investigated on the basis of the workflow implemented at CDI, by assuming as typical scenario the presence of at least three fiducial markers, correctly implanted in the lesion or in its close proximity, in order to track rotations. The presence of multiple lesions (≤3) to be treated with multiple fractions and in distinct treatment plans (e.g. metastatic patients) was also considered in the risk analysis;
- treatment delivery to spine lesions by using XSTS. The analysis of this stage was carried out considering the process implemented at the Carlo Besta Neurological Institute Foundation IRCCS.

In the second step of the FMEA, the potential failure modes, together with their causes and effects, were identified. Three indexes were then assigned to each failure mode: the occurrence rating (O), the severity rating (S), and the detectability rating (D). A ten-point scale was used to score each category, ten being the number indicating the most severe, most frequent, and least detectable failure mode respectively. The strategies and solutions currently applied at the two CyberKnife® Centres to mitigate the risk in the routine clinical practice, as well as the quality assurance practices and protocols, were taken into account in the assessment of those indexes. The risk probability number (RPN) was then calculated as the product of the three scores: RPN = O × S × D and subsequently used to rank the various failures in order of importance. The ranking scales adopted by Perks and colleagues [17] were used as guidelines. Finally, novel solutions in addition to the safety measures and strategies already adopted have been proposed to increase patient safety. In particular, the failure modes with the highest overall risk (RPN value ≥ 80), and the failures which could lead to severe injuries to the patient (severity index S ≥ 9), independently of the RPN value, were taken into account for safety improvement. We would like to point out that, because of the subjective nature of the analysis, the chosen values should not be regarded as absolute and hard thresholds, but as practical tools to identify the weakest steps of the workflow that take priority over the others.

## Results

**Fig. 1:**
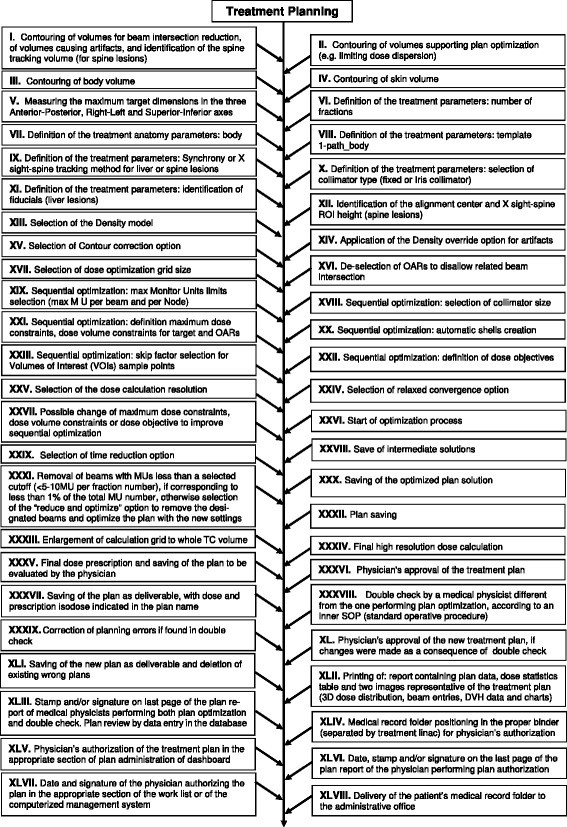
Sub-processes of the treatment planning stage in the CyberKnife® SBRT

The process-tree characterising the SBRT treatment planning stage was composed with a total of 48 sub-processes, as shown in Fig. 1. Similarly, 42 sub-processes were identified in the stage of delivery to liver tumours and 30 in the stage of delivery to spine lesions.

All the sub-processes were judged to be potentially prone to one or more failure modes. The 5 most important failures occurring in the planning stage are summarized in Table 1. Similarly, the main failure modes (in terms of overall risk or severity of the potential effects), identified in the stages of delivery to liver and spine lesions are shown in Table 2 and Table 3, respectively.

**Table 1:** FMEA of the treatment planning stage. Failures with RPN ≥ 80 or S ≥ 9 are listed

Sub-process	N	Potential failure mode	Potential causes of failure	Potential effects of failure	S	O	D	RPN
VI. Definition of the treatment parameters: number of fractions	1	Typing of a wrong number of fractions	Erroneous identification of the fractions number on the patient’s record, wrong patient’s record (coincidence of names), wrong typing	Wrong fraction dose administration	10	2	3	60
XII. Identification of the align centre and X sight-spine ROI height (in the case of spinal lesions)	2	Wrong positioning of the align centre and ROI height	Inexperience, presence of multiple lesions, damaged vertebrae	Tracking non-representative of the lesion’s movement (underdosage of the PTV, overdosage of the OAR)	7	2	7	98
XXXIII. Enlargement of the calculation grid to all the CT volume in the three views	3	Missed enlargement of the calculation grid to all the CT volume	Inexperience, distraction, haste, activity interruption	Missed visualization of the hot spots in areas far from target and OARs, partial evaluation of the DVH	9	2	3	54
XXXVI. Physician’s approval of the treatment plan, with eventual re-prescription of dose and number of fractions	4	Missed or wrong re-prescription of dose or number of fractions	Inexperience, distraction, haste, activity interruption, high workload, missed communication between physicist and physician	Erroneous dose delivery	10	2	4	80
XLII. Print of the report containing plan data, of the dose statistics table and of two images representative of the treatment plan (3D dose distribution, beams entry, DVH data and charts)	5	Missed or wrong printing of the plan report, of the table and images, printing of report, table and images not concerning the approved plan	Inexperience, distraction, haste, activity interruption, high workload, printing performed not contextually with the plan approval, missed communication among physicists	Missed check of the treatment plan, delivery of a sub-optimal plan or erroneous dose (in case there are other deliverable plans present)	10	1	4	40

**Table 2:** FMEA of the stage of delivery to liver lesions. Failures with RPN ≥ 80 or S ≥ 9 are listed

Sub-process	N	Potential failure mode	Potential causes of failure	Potential effects of failure	S	O	D	RPN
IX. Patient’s instruction on how to request the intervention of the technician in case of need (voice call via intercom and/or lifting a hand)	1	Absent or insufficient patient’s information on the request for help in case of need	Negligence, difficult communication with the patient, inattention, haste *(intensive scheduling)*	Lack of assistance in case of need, discomfort to the patient	10	1	3	30
XVI. Verifying the right vision of the patient from the control room with swiveling cameras	2	Failure to verify the vision of the patient from the cameras, suboptimal patient’s vision	Negligence, inattention, haste *(intensive scheduling)*, superficiality, cameras not working, presence of objects in the treatment room that limit the vision of the patient	Lack of monitoring (i) possible collisions between the treatment manipulator and the patient; (ii) the patient’s welfare; (iii) possible collisions between the treatment manipulator and any object present in the treatment room. Lack of action in anomalous situations; treatment not in accordance with the planned one; postponement of the treatment session	10	1	3	30
XVIII. Checking the correctness of patient and treatment plan data, check that the Synchrony field displays “Yes”	3	Failure to verify the patient and treatment data correctness, failure to verify that the Synchrony field is active	Negligence, inattention, haste *(intensive scheduling)*, interruption of the activity, patient clinical record not present at the time of treatment	Wrong dose delivery (in case of wrong prescription of dose or number of fractions in the planning stage), elongation of the work time, unnecessary live X-ray images acquisition, postponement of the treatment session	10	2	8	160
XXX. Selection of the appropriate size of the safety zone (small/medium/large), based on the patient’s size	4	Not appropriate selection of the size of the safety zone	Negligence, superficiality, inattention, haste *(intensive scheduling)*, wrong estimate of the actual size of the patient	Risk of collision between the treatment manipulator and the patient (if PDP alerts are ignored), elongation of the treatment time (for PDP alerts)	10	2	2	40
XXXVIII. At the end of each session, compilation of the specific section in the worklist by the technician who delivered the treatment	5	Missed/wrong/partial/not clear compilation of the worklist at the end of each session	Negligence, inexperience, inattention, haste *(intensive scheduling)*, interruption of the activity, patient clinical records not present at the end of the treatment, shift of technicians during the treatment (high workload)	Incorrect delivery of treatment plans (wrong plan, wrong day,…) if multiple lesions (plans) are present, incomplete patient clinical records, slowdown of the workflow.	8	2	5	80

**Table 3:** FMEA of the stage of delivery to spine lesions. Failures with RPN ≥ 80 or S ≥ 9 are listed

Sub-process	N	Potential failure mode	Potential causes of failure	Potential effects of failure	S	O	D	RPN
I. Call of the patient in the waiting room	1	The patient is called but a different one answers/ The patient is not called	Identification does not include patient’s name, surname, date of birth, photo-Patient was not informed of modifications regarding the time of the appointment, patient is late	Delivery of the treatment to the wrong patient -the radiotherapy treatment is not delivered or is administered late	10	1	2	20
II. Verification of the patient’s identity at the treatment’s room entry by asking personal data confirmation	2	Patient’s identity verification by checking all the personal data not performed	Only patient’s surname check	Possibility of mistaking patients and therefore treatments	10	2	3	60
X. Check of the correct view of the patient from the treatment workspace using adjustable video cameras	3	Patient is not monitored during treatment	Video cameras are not correctly oriented or functioning	Cyberknife may hit the patient without the operator noticing it. Patient may be in need and not been seen	9	2	2	36
XII. Patient selection using personal data (Name and surname)	4	Wrong patient’s name-Personal data check is not performed	Patient is called without checking patients’ list-Lapse of memory	Delivery of the treatment to the wrong patient-possibility of mistaking patients and therefore treatments	10	2	5	100
XIII. Check of the correct treatment plan and of the number of fractions as described on the report print	5	Delivery to the patient of a wrong plan-plan check not performed	Personal data and patient ID on the printed plan not checked-lapse of memory	Patient receives wrong irradiation-possibility of mistaking patients and therefore treatments	10	2	3	60
XV. Check of patient’s name, surname and medical ID by flagging the appropriate box for acceptance	6	Patient’s personal data not checked	Automatic action- Lapse of memory	Wrong patient or treatment-possibility of mistaking patients and therefore treatments	10	2	7	140
XVI. Check of: plan name, tracking method (XSight spine), path, number of fraction, collimator type and aperture-flag of the appropriate box for acceptance	7	Data check is wrong or not performed	High workload-lapse of memory	Wrong patient or treatment-possibility of mistaking patients and therefore treatments	10	2	7	140
XVII. Accurate alignment of the patient by comparing DRR and live images: adjustment of the values and tolerance levels defined in the image parameters window-adjustment of the X Sight Spine ROI dimensions	8	Wrong alignment-Threshold levels of the different parameters not modified when necessary	Difficulty to visually identify spine tract in the live images-Lapse of memory, insufficient experience of the operator with the treatment system	Treatment not properly delivered-longer time to start treatment	10	1	4	40
XIX. Setting of the most appropriate patient size	9	Appropriate patient size not set	Lapse of memory, insufficient experience of the operator with the delivery system	Possible collisions or errors of the PDP system slowing down treatment	9	2	5	90

## Discussion

### Planning stage

The failures N. 1, 3 and 5 (Table 1) concern overdosage and underdosage of target and OARs, and may therefore potentially lead to severe patient injury or death. Their overall risk can be considered moderate since the safety measures already adopted are deemed to guarantee a sufficient level of failure detection and/or a low frequency of occurrence. An example is the presence of a second independent global check of the approved treatment plan by a medical physicist different from the planner (second physicist check). In addition, the practice of including the number of fractions in the approved treatment plan’s file name and in the patient’s clinical record documentation (on the first page and in other dedicated sections of the worklist) proved to be effective.

However, in consideration of the high severity rating assigned, supplementary additional safety measures have also been evaluated by the working group. As an example, in order to reduce the risk of failure N.5 a clear management of multiple treatment plans for the same patient was established by avoiding the presence, at the same moment, of more than one deliverable plan. In this way, only one plan will be visible to the radiation therapist at the delivery station. Besides, the verification of the presence of deliverable treatment plans different from that approved by the physician, including even old treatment plans not completed for any reason, was included in the second independent check.

In general, the improvement of patient safety may derive from an enhancement in the communications among the staff involved in the process, and from the correct and clear traceability of the various actions. Prompt communication becomes extremely important when changes in some treatment parameters are introduced during the RT workflow, in particular regarding number of fractions and prescribed dose. Indeed, if these modifications are not properly taken into account, serious consequences for the patient can be expected. Failure N.4, i.e. missed or wrong re-prescription of dose or number of fractions, represents a clear example of this event. In order to minimize the possibility of administering a wrong dose to the patient, as mentioned in failure N.4, several corrective actions have been introduced in the workflow. As a general approach, the printing of the report of the approved plan is physically attached to the patient documentation, in order to facilitate the quick review of the correctness of the data. As a corrective action, it has been established that the physician confirms the dose prescription (number of fractions and total dose) during the evaluation of the treatment plan with the medical physicist and consequently places his signature in a specific section of the patient’s clinical record’s documentation.

The remaining relevant potential failure mode in the planning stage, consisting in the wrong positioning of the alignment centre (failure N. 2, RPN = 98), was characterized by a low detection probability. On the basis of this result, the double check of this parameter, initially not contemplated in the already adopted safety measures, was accordingly implemented.

### Delivery stage

A preliminary consideration, valid for the two Cyberknife® Centres involved in the study, is that the potential effects of FMEA on the patient’s safety do not cover only radiation protection aspects. The execution of SBRT treatments with Cyberknife® involves the movement of a heavy robotic manipulator on a virtual sphere centred on the lesion, so the risk of mechanical collision and consequent severe injury for the patient is not negligible. In the delivery stages listed by both Centres (Tables 2 entries N. 1, 2 and 4 and Table 3 entries N. 3 and 9), the importance of patient instruction on how to call for help in case of need, of the correct vision of the patient from the control room with swiveling cameras and of the selection of the appropriate size (small, medium or large) for the patient’s safety zone tool is therefore highlighted through a high severity score (S = 9, 10).

The intrinsically high detectability of these specific failures can be further improved by the supervision of a senior operator at the beginning of each delivery session, together with the issue of an operating procedure to guide the therapist in the main steps of the treatment setting. The periodic verification of the proper functioning of the devices monitoring patients welfare (intercom and cameras) is also recommended.

As far as the CDI centre is concerned, the higher RPN values in Table 2 are for failure mode N.3 (RPN = 160) and N.5 (RPN = 80). The highest RPN value is related to the missed check of patient and treatment plan data during the loading of the treatment file. Even if the double check operated by a medical physicist has greatly increased the probability to detect wrong plan data in the planning stage, the radiation therapist represents the final check of the plan’s correctness. For this reason, it is advisable to consider the independent double check of the patient and treatment data by two operators also for every first session as safety measure.

The second highest RPN (Table 2, N.5) concerns the necessity of clearly establishing the management of multiple deliverable plans, as previously stressed in the planning stage failure analysis. The presence of multiple plans at the treatment console derives from the treatment of multiple lesions in the liver, all scheduled on a short period of time. The simultaneous planning of multiple lesions is recommended to evaluate the cumulative dose received by organs at risk. Each deliverable plan can be arbitrarily selected by the operator for delivery and, if clear information about treatment schedule is not provided, incorrect delivery of treatment plans (Table 2, N.5) might be performed (e.g. not following the fractionation scheme).

The checking of the presence of one deliverable plan at a time, performed by the physicist at the planning stage, could be an appropriate measure to assure a low occurrence of this potential effect. In addition, the rather low detectability of this failure could be increased by ensuring that the therapist always warns the physicist if there is more than one deliverable plan per patient on the treatment console.

It can be noted that none of the main failures of Table 2 is directly related to the tracking procedure in spite of the fact that the Synchrony tracking system is really complex and laborious, and influences the overall quality of the treatment. In fact, the related most critical sub-processes deal with: the proper detection of the breathing waveform, the correct identification of the target (namely of the fiducials) on each live X-ray image and the accuracy of the Synchrony correlation model both in the set-up phase and throughout the treatment. The severity and detectability of the potential failure modes occurring during these sub-processes was evaluated to be medium and high (S ≤ 8, D ≤ 4) respectively. Indeed, it must be considered that the main parameters of the SRTS (i.e. radial correlation error, rigid body error, uncertainty value of the fiducials extraction algorithm) are continuously available to the operator by means of display graphs. Moreover, they are characterised by a maximum pre-set threshold value that the therapist cannot overcome. The threshold value is a software tool helping to prevent errors, alternative to the double check performed by a second operator. Accordingly, the overall risk of the potential failures related to the Synchrony tracking procedure can be assessed not significant (RPN < 80), as long as the therapists have been accurately trained in order to successfully deal with these tools.

In treatment delivery to spine lesions the potential failure modes, causes and effects for Besta Institute are reported in Table 3. The first two entries of the table concern patient call and identification by the radiation therapist. The potential detriment in these steps lies in the fact that an erroneous identification would lead to the delivery of a high dose treatment (according to the fractionation scheme) to the wrong patient and possibly to healthy tissue. A routine practice has therefore been introduced, according to which identity is being verified by the radiation therapist by letting the patient itself state personal information (such as birth date) immediately after entering the treatment area. This, together with the availability of photos in the patient’s digital record, decreases failure detectability score and consequently reduces RPN.

Failure modes four to seven all deal with verification of patient’s personal and treatment plan information (such as patient’s name and surname, plan name, number of fractions, collimator type and size etc.). At this stage, the Cyberknife® system provides the radiation therapist with many checkpoints to be ticked off. The highest RPN in Table 3 is associated with these steps of the delivery process for spinal lesions, mainly because of the high severity score (S = 10) assigned to potential radiation protection effects (patient receives wrong irradiation). Probability of detecting for these failures is also deemed to be low. Potential causes of the failures have been usually identified with the operator having a lapse of memory or dealing automatically with the procedure. For this reason, implemented corrective actions are mainly addressed to the radiation therapist and consist in: inhibition to start delivery if a printed copy of the treatment plan is not available for double check, speaking out loud treatment information while ticking them off and attendance to periodic courses on the subject.

Entry N. 8 deals with accurate alignment of the patient on the treatment couch by adjusting image parameters. Severity score for this entry was rated 10 since a poor choice of the imaging parameters might lead to the identification and treatment of an erroneous spine tract. Also, possibility to deliver an approved plan to the wrong patient cannot be excluded since, a priori, different spine tracts could be matched within imaging parameter threshold values. The most sensitive of these parameters is the *False Node Threshold.* A node is identified as a false node if no correlation is found between the Live X-ray and DRR images. A default threshold for the allowable percentage of rejected node candidates in the ROI is pre-set by the system at 50 %, but as a precaution the radiation therapist tries to keep it around 15 % or less, an higher value being an alert for further investigations. Further safety measures helping to keep a good detectability are the presence of a more expert radiation therapist taking part in the alignment procedure as a support in case of unclear or damaged vertebral anatomy or uncertain localization. Furthermore the correct matching of DRR and live images is validated by a physician before starting each treatment fraction.

In the failures analysis relative to the delivery stage for liver treatments the possibility to deliver an approved plan to a wrong patient was intentionally excluded. The tracking algorithm assesses the correspondence between the marker’s position in the DRR and in the live images with the *Rigid Body Error* parameter: a high value indicates a different configuration of the fiducials in the live images, probably due to a migration or to a wrong identification. The maximum value allowed for this parameter is 5 mm but the tracking reliability is judged as acceptable up to 3 mm. Basing on the assumed scenario of at least three fiducial markers implanted in lesion or in its close proximity and on the adoption of a good practice (Rigid Body Error < 3 mm) the authors are deeply confident that a plan cannot be delivered to a wrong patient, since three fiducials could not be implanted with the same rigid body configuration on different patients. This conclusion is valid only for at least three fiducial markers.

In addition to the failure modes specifically identified in the workflow of the two Cyberknife® Centres, a careful consideration about the aspect of traceability in SBRT treatments is required. As already mentioned in this work, it is not uncommon for one patient to receive two or more SBRT treatments over few months or years. Indeed, many treatments are performed on multiple lesion sites or on metastases that can recur either locally or in other regions of the spine or liver. In such events, traceability and record keeping are essential in order to guarantee patient safety in case a lesion is re-treated or different sites are irradiated in subsequent steps. Each Cyberknife® system is equipped with a Cyberknife® Data Management System (CDMS), which provides the storage of Cyberknife® System patient, user and system data, as well as applications and interfaces to access, add, modify, export, delete, generate reports and validate data. The plan administration tasks for the data management system contain tools enabling the user to perform administration tasks on the displayed list of active patients with their associated treatment, Quality Assurance and simulation plans. However, although patient records are present, the CDMS cannot in general be considered as a complete Record and Verify (R&V) system, due to two main shortcomings. The first one is that it is not provided with the possibility of inserting a treatment strategy (e.g. dose per fraction, fraction schedule etc.), to be compared to the one sent by the TPS for independent verification and approval.

Second point is that the CDMS does not allow the storage of a shared complete set of dosimetric information if multiple treatments to the same patient have been administered with different Linacs, even if they are all Cyberknife systems. Availability of the complete medical record in a dedicated computerized management system would allow for proper evaluation of the dose already delivered. This would provide the physician and medical physicist with all the appropriate information for a new dose prescription and dose-volume limits for the OARs, thus consistently reducing the risk of undesired and potentially dangerous overdose to healthy structures.

If a fully integrated R&V is not available all patient medical records should be manually registered at least in one computerized system or in paper format (including the dosimetric data), in order to be always available at least at the first clinical visit. Being critical and time consuming, this practice cannot prescind from an adequate staff presence.

## Conclusions

The multi-institutional application of FMEA to the planning and delivery stages in SBRT performed with CyberKnife® led to the identification of the various potential failure modes. Their analysis allowed to enhance the safety strategies already adopted in the clinical practice with additional measures for optimizing quality management workflow and increasing patient safety.

Some of the new solutions are specifically related to the CyberKnife® treatments; others are common to previous FMEA analyses [10–13] and confirmed the soundness of the general lessons and recommendations for preventing accidental exposures in the modern radiation therapy [9]. In particular, from this study came out that the competence and skill of the staff dealing with the workflow, together with a systematic double check of the main critical parameters of the process, play a decisive role for the patient safety and treatment quality. Therefore, the establishment of dedicated training schemes on the operations and limits of the tools and software employed in the RT process, as well as on the related procedures and protocols, may drastically contribute to reduce the frequency of failures and, consequently, the overall risk of accidents. Finally, excessive workload and haste should be avoided, and the work environment should encourage working with awareness, avoid distractions and facilitate concentration.

## Abbreviations

SBRT: Stereotactic Body Radiation Therapy
FMEA: Failure Mode and Effects Analysis
CDI: Centro Diagnostico Italiano
SRTS: Synchrony® Respiratory Tracking System
XSTS: Xsight® Spine Tracking System
TPS: Treatment planning system
DRR: Digitally Reconstructed Radiograph
OAR: Organs At Risk
CDMS: Cyberknife® Data Management System
RPN: Risk Probability Number
RT: Radiation Therapy
ROI: Region of interest
DVH: Dose-volume histogram
MU: Monitor unit
PTV: Planning Target Volume
